# Flunarizine as a potential repurposed drug for the serotonin transporter inhibition: an integrated approach for therapeutic development against major depressive disorder

**DOI:** 10.3389/fphar.2025.1599297

**Published:** 2025-05-13

**Authors:** Abdelbaset Mohamed Elasbali, Ahmed S. Ali, Taj Mohammad, Mohd Adnan, Anas Shamsi, Md. Imtaiyaz Hassan

**Affiliations:** ^1^ Department of Clinical Laboratory Science, College of Applied Medical Sciences-Qurayyat, Jouf University, Qurayyat, Saudi Arabia; ^2^ King Salman Center for Disability Research, Riyadh, Saudi Arabia; ^3^ Department of Physical Therapy and Health Rehabilitation, College of Applied Medical Sciences, Jouf University, Qurayyat, Saudi Arabia; ^4^ Centre for Interdisciplinary Research in Basic Sciences, Jamia Millia Islamia, New Delhi, India; ^5^ Department of Biology, College of Science, University of Ha’il, Ha’il, Saudi Arabia; ^6^ Centre of Medical and Bio-Allied Health Sciences Research, Ajman University, Ajman, United Arab Emirates

**Keywords:** major depressive disorder, serotonin transporter, drug repurposing, virtual screening, molecular dynamics simulation, flunarizine

## Abstract

Major depressive disorder (MDD) is a serious neuropsychiatric condition that affects millions of people worldwide, causing significant psychological distress and lifestyle deterioration. The serotonin transporter, which plays a critical role in regulating the uptake of serotonin (5-HT) back into presynaptic cells, is a primary target for antidepressants. Though selective serotonin reuptake inhibitors (SSRIs) are still the pharmacologic treatment of choice, alternative methods remain in demand to enhance the efficacy of treatment and offer more therapeutic options. Drug repurposing provides an efficient solution to speed up antidepressant research because it identifies existing FDA-approved medications that might inhibit the serotonin transporter. A virtual screening method was integrated into the study that examined 3620 FDA-approved drugs to discover new repurposed serotonin transporter-inhibiting molecules. The binding affinity, structural stability, and inhibitory potential were assessed using molecular docking and molecular dynamics (MD) simulations. Among the screened compounds, Flunarizine, a well-known calcium channel blocker, emerged as a promising serotonin transporter inhibitor due to its strong and stable binding configuration within the transporter’s active site. Detailed molecular docking studies revealed that Flunarizine formed key interactions with critical residues of the serotonin transporter, suggesting its potential as an effective modulator. Subsequent 500-nanosecond MD simulations further confirmed the stability of the serotonin transporter-Flunarizine complex, demonstrating minimal structural deviations and maintaining crucial dynamic properties throughout the simulation trajectory. These findings highlight Flunarizine’s potential for repurposing as a novel therapeutic agent targeting serotonin transport modulation. The study provides a solid foundation for further preclinical and clinical investigations into the antidepressant repurposing of Flunarizine.

## 1 Introduction

Major depressive disorder (MDD) is a serious, chronic neuropsychiatric condition that impacts millions of people worldwide by creating extensive emotional regulation problems, cognitive deficits, and life quality deterioration (Cui et al., 2024). It is a prevalent psychiatric condition that affects a significant portion of the population. Its lifetime prevalence ranges between 5% and 17%, with an average of approximately 12%. Notably, women are nearly twice as likely to experience MDD compared to men (Pedersen et al., 2014). The condition ranks among the significant disabilities that create substantial worldwide health problems (Yan et al., 2024). Recent epidemiological studies indicate that MDD affects over 280 million people globally, with treatment resistance rates rising to 30%–50% in the past decade (Pasman et al., 2023). According to a Global Burden of Disease (GBD) study, the COVID-19 pandemic led to an additional 53.2 million cases of major depressive disorder (MDD) worldwide, representing a 27.6% increase (Yan et al., 2024).

Patients with MDD experience ongoing depressive mood and no interest in activities (anhedonia), sleep pattern changes, appetite changes, tiredness, and persistent suicidal thoughts (Fekadu et al., 2017). The disorder results in multiple severe physiological and molecular changes because it causes alterations in neurotransmitter levels, produces neuroinflammation, and results in structural brain abnormalities (Pitsillou et al., 2020). The vague understanding of MDD pathophysiology hinders the development of effective therapeutic approaches despite researchers having studied this condition for decades. MDD exists as a complex medical condition because it develops from a combination of genetic elements, environmental triggers, and neurobiological processes, leading to the need for comprehensive treatment approaches (Marx et al., 2023).

The primary treatment approach for MDD relies on medications, and selective serotonin reuptake inhibitors (SSRIs) function as the leading prescribed antidepressants (Pehrson et al., 2015). The serotonin transporter (SERT) faces inhibition from SSRIs, which allows serotonin (5-hydroxytryptamine, 5-HT) to stay longer in the synaptic cleft (Váchalová, 2020). Scientific evidence suggests that elevated serotonin levels in the synaptic cleft through this mechanism help patients feel better and reduce their depressive symptoms (Kayabaşı et al., 2021). The medical community adopted SSRIs in the late 20th century to replace tricyclic antidepressants (TCAs) and monoamine oxidase inhibitors (MAOIs) because of their superior safety characteristics combined with lower severe side effect risks (Hengartner, 2022).

However, the widespread use of SSRIs is limited by essential drawbacks that affect both their effectiveness and patient treatment resistance (Santarsieri and Schwartz, 2015). The main disadvantage of SSRIs treatment is that patients need to take them for four to 6 weeks before they start feeling better. The prolonged therapeutic onset of SSRIs creates an essential challenge because it delays helping people who face high suicide risks (Murphy et al., 2021). The treatment resistance rate among patients who take SSRIs reaches 30%, because these medications do not provide sufficient relief for a substantial portion of patients (Howes et al., 2022). The population that shows a treatment response faces a significant risk of continued depressive symptoms even when they stay on their medications. The current treatment regime for depression requires immediate improvement through more successful antidepressants with improved treatment resistance levels.

SERT is an important protein that controls serotonin homeostasis regulation (Rudnick, 2006). The SERT protein brings serotonin back into presynaptic neurons after synaptic release, ending serotonergic signal transmission (Nichols and Nichols, 2008). The essential function of SERT in serotonin regulation makes it the primary target for antidepressant drug development (White et al., 2008). Most SSRIs function by locking onto the outward-open shape of SERT, which stops serotonin from being reabsorbed while boosting its presence outside cells (Gradisch et al., 2022). Studies indicate that SERT enters several additional structural states beyond its outward-open state, which actively controls serotonin-signaling dynamics (Kuntz, 2015). Targeting these conformations holds potential for developing new antidepressants that would be more effective and demonstrate faster antidepressant effects. The scientific community actively studies the exact mechanisms through which SERT dysregulation leads to MDD despite the prevalent use of SERT inhibitors.

Drug discovery based on conventional approaches takes a long time and resources (Doytchinova, 2022). After more than 10 years of study, developing new medicines that reach the market costs billions of dollars. Drug repurposing, also known as drug repositioning, has proven to be a successful method for speeding up new treatment development through the identification of available drugs for alternative medical uses (Kulkarni et al., 2023). Drug development costs decrease substantially when researchers use already tested medications because these drugs have previously demonstrated safety and human pharmacokinetic properties. Molecular docking and molecular dynamics (MD) simulations have become essential computational tools for drug repurposing research (Naqvi et al., 2018). Molecular docking predicts small molecules’ binding orientation and affinity to target proteins, enabling rapid virtual screening of large compound libraries (Naqvi et al., 2018). MD simulations extend these insights by modeling atomic-level movements over time (Naqvi et al., 2018).

Complementary approaches like PASS (Prediction of Activity Spectra for Substances) analysis forecast biological activities of screened molecules (Lagunin et al., 2000). At the same time, principal component analysis (PCA) and free energy landscape (FEL) calculations quantify global conformational changes and identify low-energy states linked to functional stability (Yang et al., 2009). Visualization tools like PyMOL DeLano (2002) and Discovery Studio Visualizer (2005) further dissect interaction networks, bridging computational predictions with mechanistic insights.

This study integrates molecular docking, which predicts ligand binding modes and affinities, with MD simulations that model atomic-level conformational changes over time. Together, these methods streamline drug repurposing by integrating static binding predictions, dynamic stability assessments, and functional conformation mapping.

## 2 Materials and methods

### 2.1 Molecular docking screening

A total of 3,620 compounds approved by the FDA were obtained from the DrugBank database (https://go.drugbank.com/) for the docking study (Knox et al., 2024). DrugBank was prioritized for its rigorously curated collection of FDA-approved drugs, which includes detailed pharmacological, chemical, and clinical data. The compounds were preprocessed for their structural minimization, protonation, and appropriate atom assignment types. We utilized Paroxetine as a reference molecule because it is a well-studied SERT inhibitor (Davis et al., 2016). We obtained the SERT crystal structure (PDB ID: 5I6X) from the Protein Data Bank (http://www.rscb.org) at a resolution of 3.14 Å (Coleman et al., 2016). The docking accuracy required standard protein preparation procedures, which started with solvent molecule removal followed by hydrogen atom addition and stereochemical optimization of the structure. MGL AutoDock Tools (Goodsell et al., 2021) were used to optimize the protein structure for additional optimization, culminating in energy minimization to achieve structural stability. The docking procedure was carried out using InstaDock v1.2 (Mohammad et al., 2021) in a blind search space of 86 × 67 × 83 Å grid system that concentrated at coordinates −36.109, −22.026, and 2.803 along the X, Y, and Z-axes.

The docking protocol was validated through a retrospective redocking study, in which co-crystallized Paroxetine was redocked into the SERT binding site. The redocked Paroxetine aligned closely with its original co-crystallized pose (Supplementary Figure S1), demonstrating the accuracy and reliability of the docking protocol in reproducing ligand binding conformations within the SERT pocket. The redocked Paroxetine aligned closely with its original co-crystallized pose, demonstrating an RMSD of 1.286 Å, which confirms the reproducibility and precision of the docking protocol. After the docking procedure, the binding poses were analyzed to identify compounds that showed favorable binding affinities and proper orientation and alignment with the native ligand for further evaluation.

### 2.2 Biological potential and interaction analysis

Bioinformatics technologies enable scientists to study drug-target interactions at a detailed level, thus enabling the exploration of new therapeutic ligands. The selected compounds from the molecular docking screening underwent biological evaluation through PASS (Prediction of Activity Spectra for Substances) analysis and drug profiling (Lagunin et al., 2000). The PASS web server (https://www.way2drug.com/passonline/) compares molecular structures with millions of experimentally validated bioactive molecules to determine compound biological activities. The analysis generates two probability values, Pa and Pi, where higher Pa scores indicate greater chances of biological effects occurring. A comprehensive examination of the selected compounds’ binding conformations and molecular interactions was carried out after PASS analysis. The visualization of polar interactions, hydrogen bonding, hydrophobic contacts, and π-π stacking interactions between selected molecules and SERT was performed using PyMOL (DeLano, 2002), followed by Discovery Studio Visualizer (2005) for analyzing detailed binding site molecular interactions.

### 2.3 Molecular dynamics simulations

MD simulation is a leading computational approach for researching biological macromolecules through atomic-scale modeling and conformational movement analysis (Ali et al., 2024). The technique delivers essential details about protein-ligand bindings, protein conformations, and molecular motion patterns, making it vital for contemporary drug discovery operations and biomolecular research (Mohammad et al., 2020). We utilized GROMACS 2022.3β software (Van Der Spoel et al., 2005), operated from an Ubuntu 2024 LTS system. We developed a simulation environment with precision to model molecular interactions correctly. The simulation system was hydrated using TIP3P explicit water molecules (Mark and Nilsson, 2001), without detergents or lipids. The systems were neutralized by adding suitable Na^+^ and Cl^−^ counterions. Both protein and ligand structures utilized the CHARMM36-July2022 force field (Huang et al., 2017) for calculations, while the CGenFF server (Zhu, 2019) was used to generate topology parameters for the ligands to ensure accurate force field results.

The execution of MD followed a sequence of three distinct protocols, which began with energy minimization, followed by equilibration, and finished with production simulation. The steepest descent algorithm ran for 5,000 steps to optimize the initial molecular configuration by removing steric clashes. The equilibration process occurred in two stages through NVT before transitioning to the NPT ensemble simulation. A gradual heating process from 0 K to 300 K was performed while applying weak positional restraints on the protein backbone to stop sudden structural distortions. The systems were equilibrated for 1 ns for a stable production run. The 500 ns production MD simulations enabled a complete structural dynamics analysis between free protein and protein-ligand complexes. The simulation trajectories were recorded every 10 ps to observe essential molecular interactions and conformational changes throughout the simulation period.

### 2.4 Principal component and free energy landscape analyses

Principal component analysis (PCA) is a statistical tool that discovers major motion patterns in MD trajectory data (Moradi et al., 2024). PCA of protein-ligand complexes helps to understand and visualize the system’s collective motions by reducing the data’s dimensionality (Yang et al., 2009). It helps identify key conformational changes and visualize the dynamics in a lower-dimensional space. PCA was used to investigate the atomic motion and global conformational dynamics of SERT and its drug-bound complexes. The methodology enabled the detection of necessary protein movements, alongside detecting meaningful functional protein conformations (Yang et al., 2009). The eigenvector coordinates were determined using a positional covariance matrix calculated from MD simulation data. The principal motions emerged when the covariance matrix underwent an orthogonal transformation for diagonalization to create an eigenvalue diagonal matrix.

The principal components (PCs) obtained from eigenvalues and eigenvectors represented the protein-ligand system’s most significant large-scale atomic motions. PC1 and PC2 emerged as the leading factors that explained atomic displacement. The system’s conformational transitions received deeper analysis through free energy landscape (FEL) generation. We can study protein stability and conformational states through FEL analysis by exploring free energy changes across principal components (Abdelsattar et al., 2021). This method demonstrated the capability to show stable conformational states as energy minima while identifying possible transition states involved in ligand-induced conformational changes.

## 3 Results and discussion

### 3.1 Molecular docking screening identifies potential SERT binders

Molecular docking is a standard computational approach that predicts how ligands orient and fit inside the target protein’s active sites (Naqvi et al., 2018). This methodology reveals the assessment of binding strength, interaction patterns, and modulating potential, enhancing its utility for pharmaceutical research. We used a set of 3620 FDA-approved drug molecules from the DrugBank database for virtual screening against SERT. We utilized Paroxetine, a well-known SERT inhibitor and FDA-approved SSRI, to provide reference standards for binding affinity and interaction profile assessments. The docking analysis identified the top 10 compounds by evaluating their binding energy scores, which indicate how strongly the compounds bind to SERT (Table 1). The selected molecules showed appreciable binding affinities through docking scores ranging from −10.5 to −11.2 kcal/mol, while Paroxetine exhibited a binding affinity of −8.0 kcal/mol. The strength of ligand-protein interactions during molecular docking increases as the binding energy values decrease, indicating better potential for effective inhibition (Sousa et al., 2006). These compounds show significant potential to function as competitive SERT inhibitors because they demonstrate stronger binding affinity than Paroxetine.

**TABLE 1:** List of screened hits against SERT and their docking parameters.

S. No.	Drug	PubChem ID	Binding affinity (kcal/mol)	Ligand efficiency (kcal/mol/non-H atom)	Torsional energy
1	Lidoflazine	3926	−11.2	0.3111	2.8017
2	Fentonium	10347880	−11.2	0.3111	3.113
3	Darifenacin	444031	−11.1	0.3469	2.1791
4	Bagrosin	21893707	−10.9	0.4955	0.3113
5	Flunarizine	941361	−10.9	0.3633	1.8678
6	Carindacillin	93184	−10.7	0.3057	2.4904
7	Conivaptan	151171	−10.6	0.2789	1.2452
8	Florantyrone	10617	−10.6	0.4609	1.5565
9	Talampicillin	71447	−10.6	0.3118	2.1791
10	Dabrafenib	44462760	−10.5	0.3	2.1791
11	Paroxetine	43815	−8.0	0.3333	1.2452

### 3.2 PASS analysis and drug profiling predict antidepressant potential

The PASS analysis represents a commonly utilized computational method that uses structural properties to forecast biological activity profiles of molecules (Lagunin et al., 2000). The server evaluates molecular structures against numerous bioactive compounds in databases to predict possible pharmacological results. We utilized PASS analysis to determine biological activity properties of the top 10 molecules selected from molecular docking screening against SERT. The study of ten molecules made Flunarizine stand out as the best candidate based on its drug profile associated with depression, migraine, and neuroprotective activities. The PASS results showed that Flunarizine possesses a strong potential for treating neurodegenerative and psychiatric disorders, which supports its development as an SERT inhibitor (Table 2). The PASS predictive tool generates two probability values known as Pa and Pi to assess activity potential. A compound demonstrates promising biological effectiveness if its Pa score exceeds Pi. The antidepressant and neurological disorder treatment capability of Flunarizine was confirmed through Pa values spanning from 0.618 to 0.927. However, further experimental validation of Flunarizine as a repurposed SERT inhibitor should be carried out for MDD and related neuropsychiatric conditions. The reference drug Paroxetine also showed antidepressant, nootropic, and mood disorder treatment with considerably high potential, which validated the results from the PASS predictions.

**TABLE 2:** PASS analysis of the selected molecules with their predicted activity. Pa, probability to be active, Pi. Probability of being inactive.

S. No.	Drug molecule	Chemical structure	Pa	Pi	Activity
1	Flunarizine	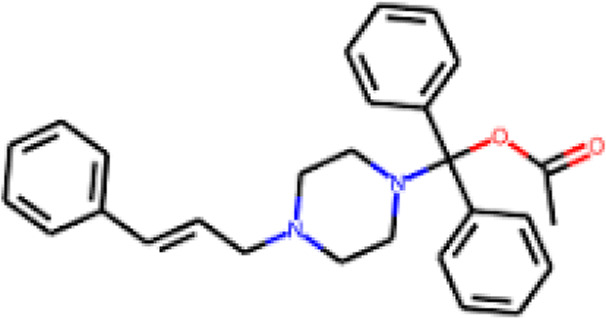	0,927	0,004	Antieczematic
			0,686	0,007	Antipsychotic
			0,609	0,036	Acute neurologic disorders treatment
			0,566	0,001	Raynaud’s phenomenon treatment
			0,618	0,114	Phobic disorders treatment
2	Paroxetine	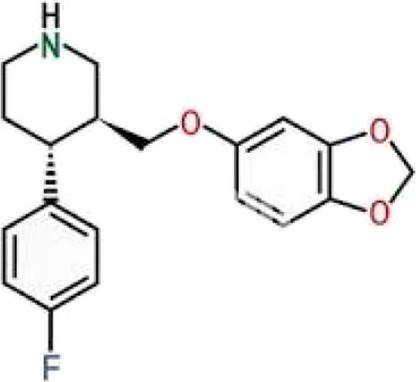	0,775	0,005	Neurotransmitter uptake inhibitor
			0,710	0,007	Mood disorders treatment
			0,667	0,009	Antidepressant
			0,684	0,045	Nootropic
			0,609	0,036	Acute neurologic disorders treatment

### 3.3 Key interactions stabilize Flunarizine in the SERT binding pocket

**FIGURE 1:**
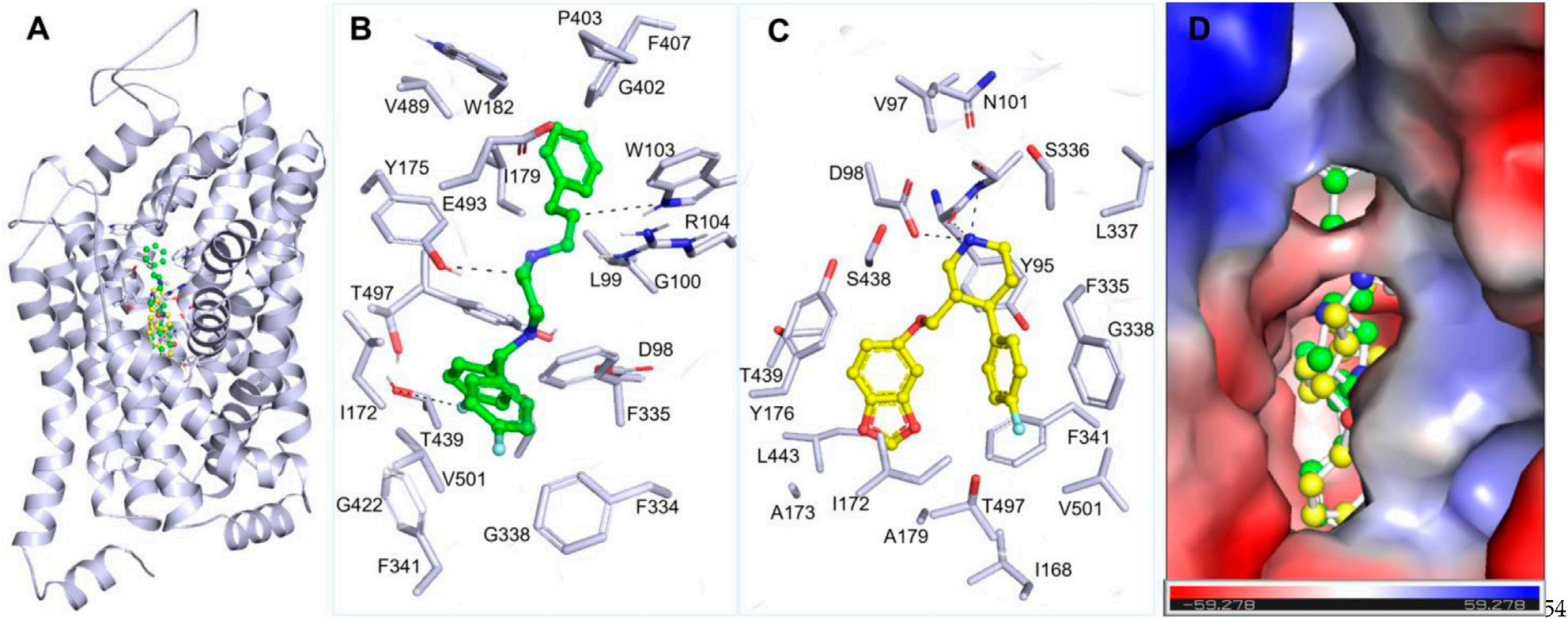
Molecular interactions of SERT with Flunarizine (green) and Paroxetine (yellow). **(A)** A 3D representation of the SERT binding pocket occupied by Flunarizine and Paroxetine. **(B)** A magnified visualization of key molecular interactions between Flunarizine and SERT binding residues. **(C)** Magnified visualization of key molecular interactions between Paroxetine and SERT binding residues. **(D)** Charged-surface illustration highlighting the electrostatic environment of the SERT binding site, demonstrating how Flunarizine and Paroxetine interact within the cavity. The figures were generated through PyMOL using the protein-ligand coordinates from the docking study.

The interaction analysis of Flunarizine and the reference inhibitor was carried out to understand their binding mechanism with SERT (Figure 1A). The study demonstrated that Flunarizine binds in the SERT active site, showing binding characteristics similar to Paroxetine (Figure 1A). Flunarizine forms various stabilizing chemical bonds with critical SERT binding pocket residues, demonstrating its strong potential to act as a promising inhibitor (Figure 1B). Similarly, Paroxetine also interacts with this binding site of SERT, which showed various common interactions (Figure 1C). The spatial orientation of Flunarizine alongside its binding conformation showed that it binds with critical function-related residues, including Thr439, a serotonin binding site (Yang and Gouaux, 2021), like Paroxetine (Figure 1D). The Flunarizine binding pattern indicates that this compound would function as an effective serotonin-competitive inhibitor of SERT by analogously inhibiting its binding site to current prescribed SSRIs.

Flunarizine establishes a promising complex with SERT through multiple hydrogen bonds combined with π-stacking and hydrophobic interactions, thus potentially increasing its inhibitory effects. Flunarizine forms various other interactions with critical residues more than Paroxetine does, demonstrating a potentially superior binding affinity and more stable interaction with SERT. The residues such as Tyr175, Ser438, Thr439, and Phe341 play essential roles in the inhibitory process by forming crucial bonding interactions that sustain SERT inhibition (Yang and Gouaux, 2021). Flunarizine’s binding interactions surpass Paroxetine’s because it establishes multiple additional interactions. The additional molecular interactions increase the binding energy, indicating that Flunarizine demonstrates a better potential for inhibiting SERT. The wide usage of Paroxetine as an SSRI reveals that Flunarizine reflects the potential to become a repurposed antidepressant drug with new applications.

**FIGURE 2:**
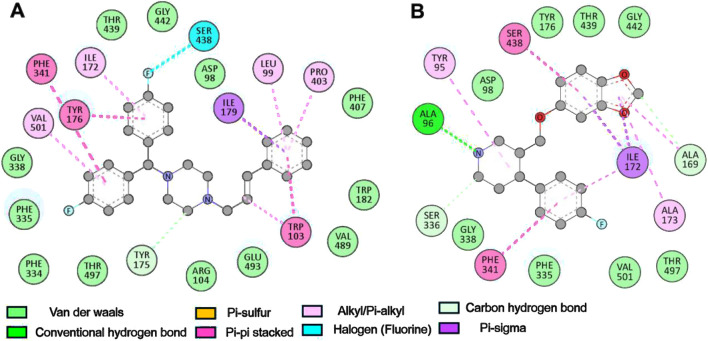
Binding site analysis of SERT-ligand interactions. **(A)** Binding residues involved in interactions with Flunarizine. **(B)** Binding residues interacting with Paroxetine. The figures showed various interactions between the protein-ligand complexes, such as hydrogen bonding, hydrophobic, and electrostatic interactions. Discovery Studio Visualizer was utilized to categorize interactions based on atomic distances (<4.0 Å for van der Waals, <3.5 Å for hydrophobic, and <3.2 Å for hydrogen bonds).

Further, a detailed analysis showed that the binding mechanism of Flunarizine with critical residues in the SERT binding cavity occurs through multiple interaction types, including hydrogen bonds, halogen bonds, π-interactions, and van der Waals contacts (Figure 2). Flunarizine establishes close interactions with the SERT binding site (Figure 2A). The ligand stability was supported by two interactions: hydrogen bonding with Tyr175 and halogen bonding with Ser438. The π-sigma interaction occurred between Ile179 and Flunarizine, while π-π stacking interactions formed with Tyr176, Trp103, and Phe341. The SERT-Flunarizine complex received stabilization from multiple hydrophobic interactions between Leu99, Trp103, Ile172, Pro403, and Val501 as well as van der Waals interactions that included Asp98, Arg104, Trp182, Phe334, Phe335, Gly338, Phe407, Thr439, Gly442, Val489, Glu493, and Thr497 (Table 3). At the same time, the reference compound Paroxetine also showed a similar interaction profile as Flunarizine (Figure 2B).

**TABLE 3:** Protein ligand interactions formed by Flunarizine and Paroxetine with SERT.

Molecule	Hydrogen bonds	Alkyl/Pi-alkyl	Van der waals interactions	Other interactions
Flunarizine	Tyr175	Leu99, Trp103, Ile172, Pro403, Val501	Asp98, Arg104, Trp182, Phe334, Phe335, Gly338, Phe407, Thr439, Gly442, Val489, Glu493, Thr497	Ser438, Ile179, Tyr176, Trp103, Phe341
Paroxetine	Ala96, Ala169, Ser336	Tyr95, Ala169, Ile172, Ala173	Asp98, Tyr176, Phe335, Gly338, Ser439, Gly442, Thr497, Val501	Ile172, Phe341, Ser438

It forms hydrogen bonds with Ala96, Ala169, and Ser336 and a single π-sigma interaction with Ile172. Additionally, Phe341 and Ser438 formed π-π stacking interactions, and Tyr95, Ala169, Ile172, and Ala173 formed alkyl/Pi-alkyl interactions. The binding of Paroxetine with SERT formed various van der Waals contacts to Asp98, Tyr176, Phe335, Gly338, Ser439, Gly442, Thr497, and Val501. Flunarizine forms a hydrogen bond with Tyr175 (2.8 Å) and a halogen bond with Ser438 (3.1 Å), residues critical for SERT’s conformational transitions. In contrast, Paroxetine interacts weakly with Ala96 (3.2 Å) and lacks halogen bonding, explaining its lower binding affinity. The analysis revealed that Flunarizine formed various common and more contact points with the serotonin binding site residues of SERT than Paroxetine, thus indicating a potentially more substantial inhibitory potential (Yang and Gouaux, 2021).

### 3.4 MD simulations confirm structural stability

The MD simulation method delivers essential information about protein and protein-ligand complexes while demonstrating structural shifts and helping to evaluate binding persistence throughout time (Xue et al., 2022). We performed an all-atom 500 ns MD simulation through GROMACS to analyze SERT and its complexes with SERT-Flunarizine and SERT-Paroxetine. The results of energy calculations showed that SERT potential energy decreased from −1,717,590 kJ/mol for SERT to −1,190,080 kJ/mol for SERT-Flunarizine and −1,190,630 kJ/mol for SERT-Paroxetine. The protein-ligand complex stability improved compared to unbound SERT, as shown by total energy values of −1,380,460, −948,548 kJ/mol, and −949,102 for SERT, SERT-Flunarizine, and SERT-Paroxetine, respectively. The binding stability of Flunarizine indicates it to be higher than Paroxetine’s, thus indicating its potential to act as a competitive SERT inhibitor. The analysis demonstrates how Flunarizine forms a solid complex with SERT, which supports its potential application as a treatment drug for MDD. Various structural deviations and flexibility parameters were analyzed from the MD simulations trajectories, as discussed in the ensuing sections.

#### 3.4.1 Stability assessment and structural dynamics

**FIGURE 3:**
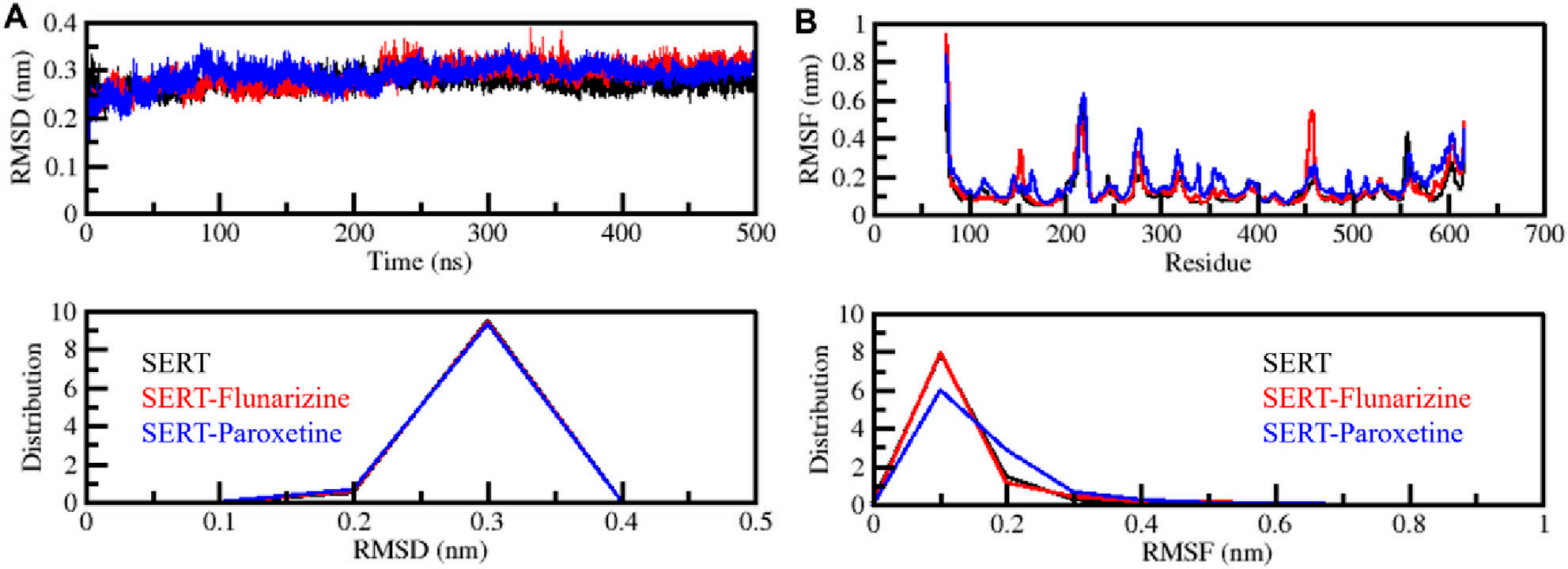
Structural stability assessment of SERT-ligand complexes. **(A)** Root mean square deviation (RMSD) plot representing the structural deviations of SERT, SERT-Flunarizine, and SERT-Paroxetine complexes over 500 ns. **(B)** Root mean square fluctuation (RMSF) plot showing residue-wise flexibility of each system. The lower panels display the distribution profiles of RMSD and RMSF values.

The structural stability of macromolecules during MD simulations can be calculated through the root mean square deviation (RMSD) measurements as an essential evaluation parameter (Kuzmanic and Zagrovic, 2010). The analysis of SERT, SERT-Flunarizine, and SERT-Paroxetine complex trajectories through RMSD evaluated their conformational changes during the simulation period (Figure 3A). The average RMSD results showed 0.27 nm, 0.28 nm, and 0.28 nm for SERT, SERT-Flunarizine, and SERT-Paroxetine, respectively. The results indicate that SERT complexes with bound ligands showed slightly higher deviations than the unbound SERT structure, indicating minor structural changes from ligand binding. The SERT-Paroxetine complex initially showed large initial deviations during the first 200 ns but stabilized afterward, whereas the SERT-Flunarizine complex exhibited constant stability without significant variations. Analysis using distribution plots showed that Flunarizine maintains SERT stability like Paroxetine during the entire simulation period (Figure 3A, lower panel). Stable ligand-protein interaction and strong binding affinity emerge from the overall lower fluctuations observed in the SERT-Flunarizine complex, which indicates its potential as a promising binder.

The root mean square fluctuation (RMSF) analysis is widely used to determine the flexibility of a protein molecule at the amino acid residue level (Khan et al., 2022). We performed RMSF analysis in SERT and its ligand-bound complexes from the simulated trajectories. The average RMSF results showed 0.11 nm, 0.13 nm, and 0.16 nm for SERT, SERT-Flunarizine, and SERT-Paroxetine, respectively. The results indicated that SERT-Flunarizine binding contributed to the stable pattern in residual fluctuations with some minor flexibility, most pronounced in the flexible loop areas (Figure 3B). The SERT-Flunarizine complex showed minimal motions during analysis, with specific stability occurring at residues 340–350 and 490–500. The similar RMSF pattern shown in the distribution plot supports the theory that Flunarizine strengthens SERT stability, which might result in prolonged inhibitory activity (Figure 3B, lower panel). Previous studies have shown that effective SERT inhibitors produce stable interactions with the protein structure (Nguyen et al., 2024). The stable nature of the SERT-Flunarizine complex indicates that Flunarizine maintains a long-lasting binding at the receptor site, which benefits antidepressant action. Further exploration of Flunarizine as a potential antidepressant drug candidate should focus on its functional impact on serotonergic neurotransmission.

#### 3.4.2 Compactness and folding mechanism examination

**FIGURE 4:**
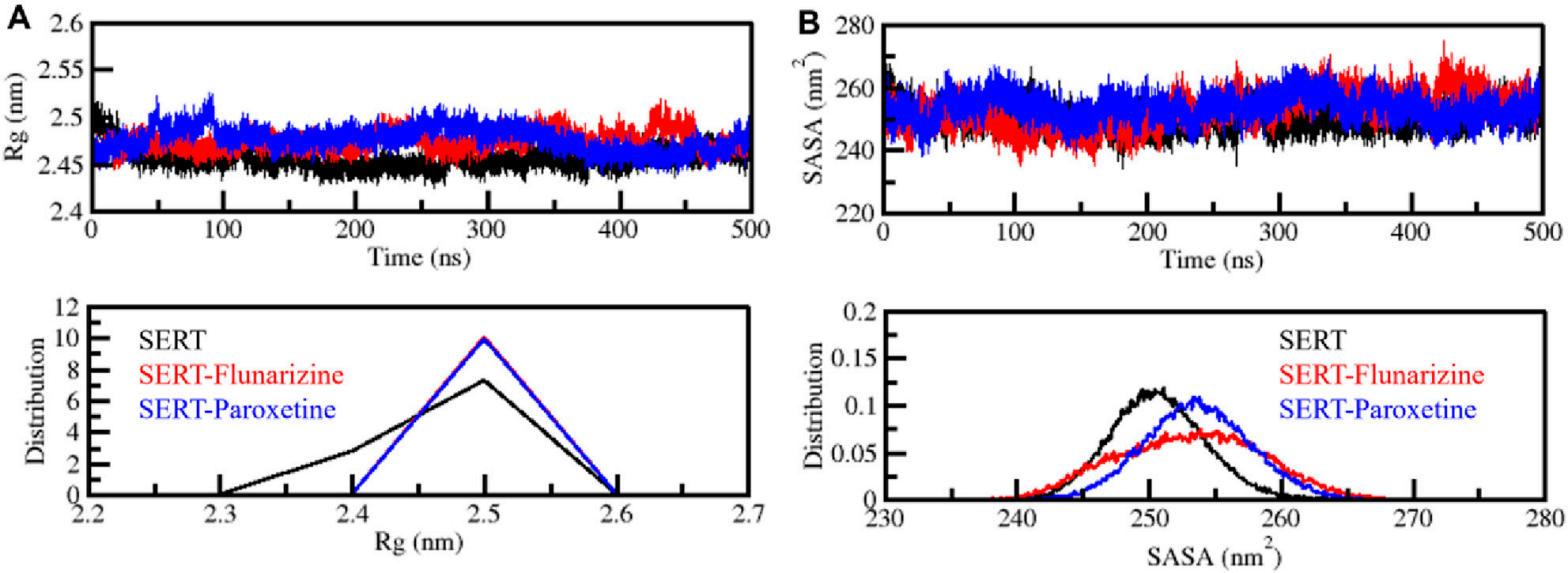
Compactness and folding behavior assessment. **(A)** Radius of gyration (*R*_g_) plot, demonstrating SERT’s compactness and structural integrity before and after ligand binding. **(B)** Solvent-accessible surface area (SASA) plot, analyzing the degree of solvent exposure for SERT, SERT-Flunarizine, and SERT-Paroxetine complexes. The lower panel illustrates the distribution profiles of *R*_g_ and SASA values.

The radius of gyration (*R*_g_) is an essential measure to assess protein and protein-drug complex folding characteristics and their compactness properties (Lobanov et al., 2008). The *R*_g_ value changes expose structural binding information because an *R*_g_ increase shows protein flexibility, while a decrease indicates stable, tight packing. We measured *R*_g_ values for SERT along with SERT-Flunarizine and SERT-Paroxetine complexes through 500 ns of simulation to evaluate protein-ligand complex compactness. The average *R*_g_ results showed 2.4 nm for each system, SERT, SERT-Flunarizine, and SERT-Paroxetine. All three systems showed almost similar average *R*_g_ values in the plot presented in Figure 4A. The *R*_g_ values of the SERT-Flunarizine complex showed lower measurements than the SERT-Paroxetine complex, with a single exception at 400–450 ns, where the values were briefly higher. This indicates that Flunarizine promotes SERT to adopt a more compact structural arrangement. The distribution plot analysis verified that Flunarizine binding produces stable protein structures by restricting protein expansion (Figure 4A, lower panel).

Protein and ligand complex structural stability and solvent exposure were examined using solvent-accessible surface area (SASA) analysis. The values of SASA directly correlate to how much protein surface area remains exposed to solvent molecules, as higher values show increased solvent contact, but lower values indicate structure compaction (Chingin and Barylyuk, 2018). The average SASA results showed 250.6 nm^2^, 253.1 nm^2^, and 253.7 nm^2^ for SERT, SERT-Flunarizine, and SERT-Paroxetine. The SASA plot indicated that the SERT-Paroxetine complex had elevated solvent accessibility at 253.7 nm^2^. In comparison, the SERT-Flunarizine complex consistently displayed reduced SASA values with an average of 253.1 nm^2^, which pointed to a compact conformation (Figure 4B). The SERT-Flunarizine trajectory showed brief solvent accessibility increases at times 410 ns and 450 ns, but immediately returned to a steady state of lower accessibility during the simulation period. The binding of Flunarizine to SERT produces a more compact receptor state that improves its stability while lowering its flexibility according to simulation results. The lower solvent exposure observed for the SERT-Flunarizine complex further supports the hypothesis that Flunarizine establishes a stable binding interaction that reduces unnecessary protein fluctuations, a desirable characteristic for effective SERT inhibition (Figure 4B, lower panel). This structural stability may contribute to prolonged receptor occupancy and improved pharmacological efficacy, reinforcing Flunarizine’s potential as a repurposed antidepressant candidate.

#### 3.4.3 Stability assessment by hydrogen bonds analysis

**FIGURE 5:**
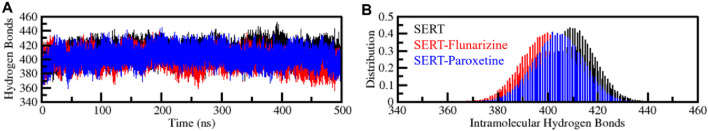
Intramolecular hydrogen bond analysis, evaluating the number of internal hydrogen bonds formed within SERT alone and SERT-Flunarizine and SERT-Paroxetine complexes. **(A)** Intramolecular hydrogen bonds formed within SERT before and after Flunarizine and Paroxetine binding. **(B)** The PDF panel represents the probability distribution function of intramolecular hydrogen bond formation over the simulation trajectory. This analysis reflects changes in protein stability upon ligand binding.

Intramolecular hydrogen bonds are essential for protein structure, structural stability, and functional conformation (Williams and Ladbury, 2003). The intramolecular hydrogen bonds of SERT were examined before and after the ligands binding (Figure 5). The hydrogen bond numbers for SERT, SERT-Flunarizine, and SERT-Paroxetine complexes were 409, 399, and 404, respectively (Figure 5A). The intramolecular hydrogen bond trajectory proved the SERT-Flunarizine complex followed the SERT-Paroxetine pattern up to 200 ns, with the same level of stability. Beyond 200 ns, the SERT-Flunarizine complex showed a slight decrease in intramolecular hydrogen bonds, likely due to the initial structural adaptation upon drug binding (Supplementary Figure S2). Intramolecular hydrogen bonds decreased marginally (399 vs. 409 in apo-SERT), reflecting local flexibility adjustments without global destabilization. Nevertheless, overall stability was preserved as the complex stayed with the native conformation throughout the simulation. The distribution plot also detected differences in hydrogen bond numbers, confirming the role of ligand binding as a stabilizer (Figure 5B). The observed reduction in intramolecular hydrogen bonding within SERT upon ligand binding may be attributed to the ligand’s occupancy of the binding pocket, which disrupts pre-existing hydrogen bonds. This disruption likely results from the ligand’s steric and electronic effects, which reorient local residues and alter hydrogen bond networks. The overall stability remained intact, as the complex retained its native conformation throughout the simulation.

**FIGURE 6:**
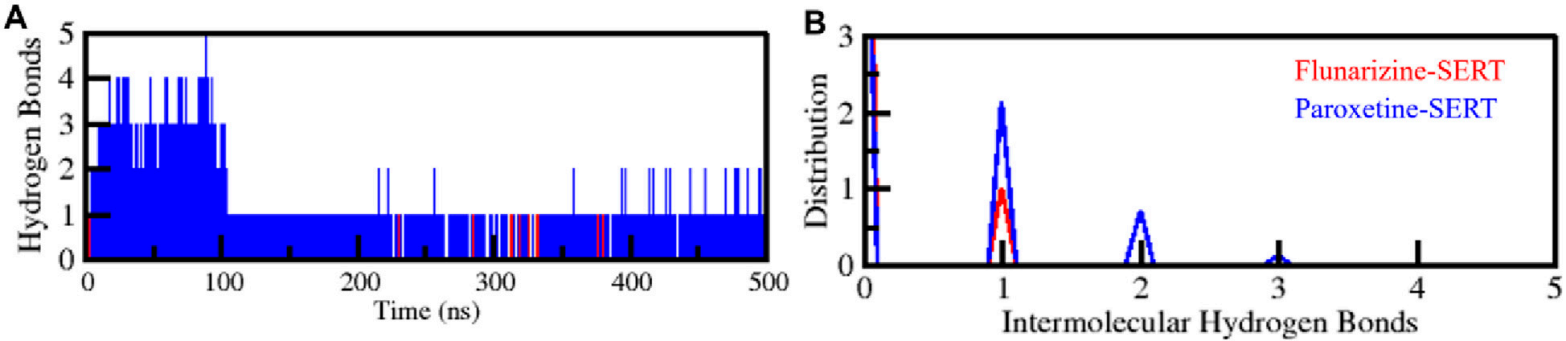
Intermolecular hydrogen bonding analysis between SERT and its bound ligands. **(A)** Hydrogen bonds formed between SERT and Flunarizine and Paroxetine. **(B)** The PDF panel represents the probability distribution function of hydrogen bond formation over the simulation trajectory.

The hydrogen bonding between the protein and the ligand is essential for drug selectivity, binding affinity, and functional efficacy (Williams and Ladbury, 2003). For the assessment of protein-ligand interaction, the intermolecular hydrogen bonds were also examined (Figure 6). It was revealed that SERT-Flunarizine and SERT-Paroxetine complexes formed one to five hydrogen bonds, respectively (Figure 6A). The distribution plot within the lower panel revealed that a minimum of one hydrogen bond was maintained through the simulation within each complex, yielding protein-ligand interaction stability (Figure 6B). The findings reveal that while Flunarizine formed fewer intermolecular hydrogen bonds than Paroxetine, it could nonetheless interact with SERT stably, with the protein not losing its original conformation upon binding. The minor loss of intermolecular hydrogen bonds for SERT-Flunarizine reflects structural adaptation upon ligand binding, common with high-affinity inhibitors (Mitra and Dash, 2018). The ligand-bound complexes of SERT were structurally stable, supporting their potential as promising binders with a well-preserved binding conformation.

#### 3.4.4 Secondary structure evaluation

The secondary structure elements of SERT were tracked to assess the structural effect of ligand binding during the simulation. Secondary structure trajectories were obtained using the DSSP (Define Secondary Structure of Proteins) program (Zacharias and Knapp, 2014) and were visualized using the XMGRACE tool (https://plasma-gate.weizmann.ac.il/Grace/). The secondary structure plot showed different structural elements represented by different colors (Supplementary Figure S3). The results showed that the SERT-Flunarizine complex had a minimal decrease in the residues in forming secondary structure elements. In contrast, the SERT and SERT-Paroxetine complexes had the same number of structured residues. Notably, the residues involved in α-helices increased minimally upon binding with Flunarizine. A minimal decrease in the number of residues involved in bends, turns, and coils was observed, which can be attributed to the introduction of local structural flexibility by Flunarizine and Paroxetine. Although these changes were minimal, SERT’s global secondary structure integrity remained unaffected after binding with Flunarizine, reflecting that the ligand does not induce significant protein destabilization. The results indicate that Flunarizine binds with SERT without perturbing the global structural conformation, which further indicates its potential as a stable and potent SERT binder.

### 3.5 Principal components analysis reveals conformational restrictions

**FIGURE 7:**
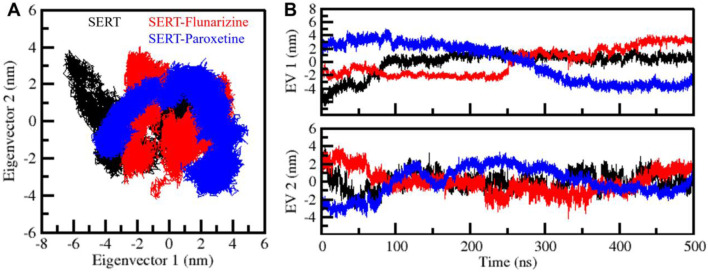
Principal component analysis (PCA) of SERT-ligand complexes. **(A)** PCA plots representing the conformational space explored by SERT, SERT-Flunarizine, and SERT-Paroxetine complexes, derived from PC1 and PC2. **(B)** Eigenvector representation, illustrating principal component fluctuations over time, provides insights into structural transitions and stabilization patterns.

PCA was employed to analyze SERT’s molecular motion, conformation flexibility, and ligand complexes (Figure 7). PCA can identify dominant motion patterns by reducing the complexity of molecular dynamics trajectories into principal components (PCs). During the simulation, the first principal components (PC1 and PC2) were employed to plot the vibration space traversed by SERT, SERT-Flunarizine, and SERT-Paroxetine complexes. The 2D subspace PCA plot illustrated the unbound SERT protein traversing a more expansive conformation space, significantly along PC2, reflecting enhanced flexibility and dynamic motion (Figure 7A). The SERT-Flunarizine and SERT-Paroxetine complexes demonstrated more constrained motion distributions along PC1, reflecting reduced conformation flexibility upon ligand binding. The motion ranges observed were PC1: −6.6–2.5 nm and PC2: −2.9–3.8 nm for SERT, PC1: −2.8–4.1 nm and PC2: −4.2–3.9 nm for SERT-Flunarizine, and PC1: −4.8–4.9 nm and PC2: −4.0–3.4 nm for SERT-Paroxetine. To further analyze the conformational state stability, eigenvector plots against time were made for the three systems (Figure 7B). The observation demonstrated SERT with prominent structural fluctuations, while SERT-Flunarizine and SERT-Paroxetine complexes assumed different and stable conformations with time. The stabilization of PC1 and PC2 fluctuations with time reflects the inhibition of excessive protein motion upon ligand binding and the stabilization of SERT into a structured conformation. The results confirm the orientation of a stable conformational state by Flunarizine comparable with Paroxetine, further establishing its potential as a SERT inhibitor.

### 3.6 Free energy landscape analysis reveals conformational folding

**FIGURE 8:**
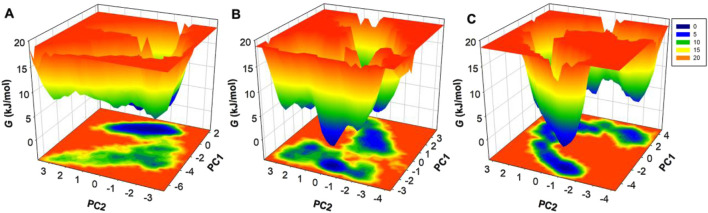
Free energy landscape (FEL) analysis of SERT conformational states. **(A)** Three-dimensional Gibbs free energy map of unbound SERT, showing its native state energy basin. **(B)** FEL map of SERT-Flunarizine complex. **(C)** FEL map of SERT-Paroxetine complex. Areas represented as a dark blue basin correspond to low-energy conformations near the native state.

FEL analysis was performed to investigate further the thermodynamic stability and conformation transitions of SERT and its ligand-bound complexes. FEL maps were obtained from PC1 and PC2 from PCA trajectory data and yielded insights into transition states from metastable towards the native conformation. The 3D Gibbs FEL maps depict changes in energy across conformation states. The areas represented in red correspond to high-energy unstable states, and the regions described in dark blue correspond to low-energy conformations (Figure 8). The unbound SERT protein displayed a broad dark blue basin corresponding to a dynamic and flexible native conformation (Figure 8A). The SERT-Flunarizine complex displayed tighter single dark blue basins corresponding to the constraining of the conformation space by the ligand and stabilization of SERT into singular low-energy conformations (Figure 8B). The SERT-Paroxetine displayed a wider low-energy state confined within multiple basins (Figure 8C). Here, metastable states were also observed in both ligand-bound complexes, corresponding to minor conformation adjustments upon the binding of the ligand. Taken together, the ligand-bound SERT in the presence of Flunarizine exhibited stability in MD simulations and essential dynamics. These findings suggest that Flunarizine can be further explored experimentally as a potential repurposed drug targeting SERT inhibitors for therapeutic development against MDD.

Overall, the results of this study demonstrate that a calcium channel blocker, Flunarizine, showed promise as an antidepressant drug that can be repurposed to target SERT. SERT regulates synaptic serotonin levels, and its dysregulation is implicated in MDD’s emotional and cognitive deficits. Flunarizine is a selective channel entry blocker with calmodulin binding properties and histamine H1 blocking activity (Stubberud et al., 2019). It is well-established that calcium channel blockers have been explored for their potential antidepressant effects (Harrison et al., 2020; Bozorgi et al., 2020). By competitively inhibiting SERT, Flunarizine prolongs serotonin availability, mirroring SSRIs’ mechanism but with enhanced binding stability. The research uses computational screening technology to show how existing FDA-approved drugs can be evaluated for new pharmacological purposes, shortening the drug discovery timeline. While docking identifies initial hits, MD simulations validate their dynamic stability, a critical step absent in standalone docking studies. This synergy mitigates false positives and confirms Flunarizine’s binding persistence. Recent studies corroborate the utility of integrated computational approaches in small-molecule inhibitor discovery (Vikhar Danish Ahmad et al., 2024). Further experimental evaluations on Flunarizine must be performed to verify computational results and assess the drug’s effectiveness for MDD treatment. Future work should prioritize *in vitro* assays (e.g., serotonin uptake inhibition in neuronal cell lines) and *in vivo* behavioral studies in rodent models of depression to validate Flunarizine’s antidepressant effects. Additionally, pharmacokinetic studies and safety profiling are essential to assess its suitability for repurposing in MDD. These steps will bridge the gap between computational insights and clinical application.

## 4 Conclusion

This study utilized an integrated computational approach to identify FDA-approved drugs as potential SERT inhibitors for repurposing in MDD. Virtual screening of 3,620 FDA-approved drugs from the DrugBank library ranked Flunarizine ahead of other drugs based on its higher binding affinity (−10.9 kcal/mol) towards SERT, surpassing the comparative reference SSRI Paroxetine (−8.0 kcal/mol). Molecular docking disclosed key stabilizing contacts of Flunarizine with SERT’s active site, such as hydrogen bonds, π-π stacking, and hydrophobic contacts with residues critical for serotonin uptake. All-atom MD simulations for 500 ns proved the structural stability of SERT-Flunarizine, as indicated by minimal fluctuations in RMSD values (0.28 nm), decreased residual flexibility (RMSF = 0.13 nm), and compact folding (*R*_g_ = 2.4 nm). PCA and FEL calculations further verified that Flunarizine limits SERT to a low-energy conformational state, effectively replicating the inhibitory action of SSRIs but with better dynamic stability. Flunarizine’s dual action as a calcium channel blocker and SERT inhibitor can make it a multi-target drug candidate for MDD, consistent with new evidence on the involvement of calcium signaling in neuropsychiatric illness. These computational predictions indicate repurposing possibility, confirmation of SERT inhibition efficacy and tolerability by preclinical validation using cellular and animal models is essential. While our findings highlight Flunarizine’s promise, translational validation through preclinical and clinical studies remains imperative to establish its efficacy and safety for MDD treatment.

## Data Availability

The original contributions presented in the study are included in the article/Supplementary Material, further inquiries can be directed to the corresponding authors.
