# Supplementary material for: Flunarizine as a potential repurposed drug for the serotonin transporter inhibition: an integrated approach for therapeutic development against major depressive disorder
Source: Front Pharmacol. 2025 May 13;16:1599297. doi: 10.3389/fphar.2025.1599297 (PMC12120357; doi:10.3389/fphar.2025.1599297)
Supplement: Supplementary file 1 [file Presentation1.pptx]

## Slide 1
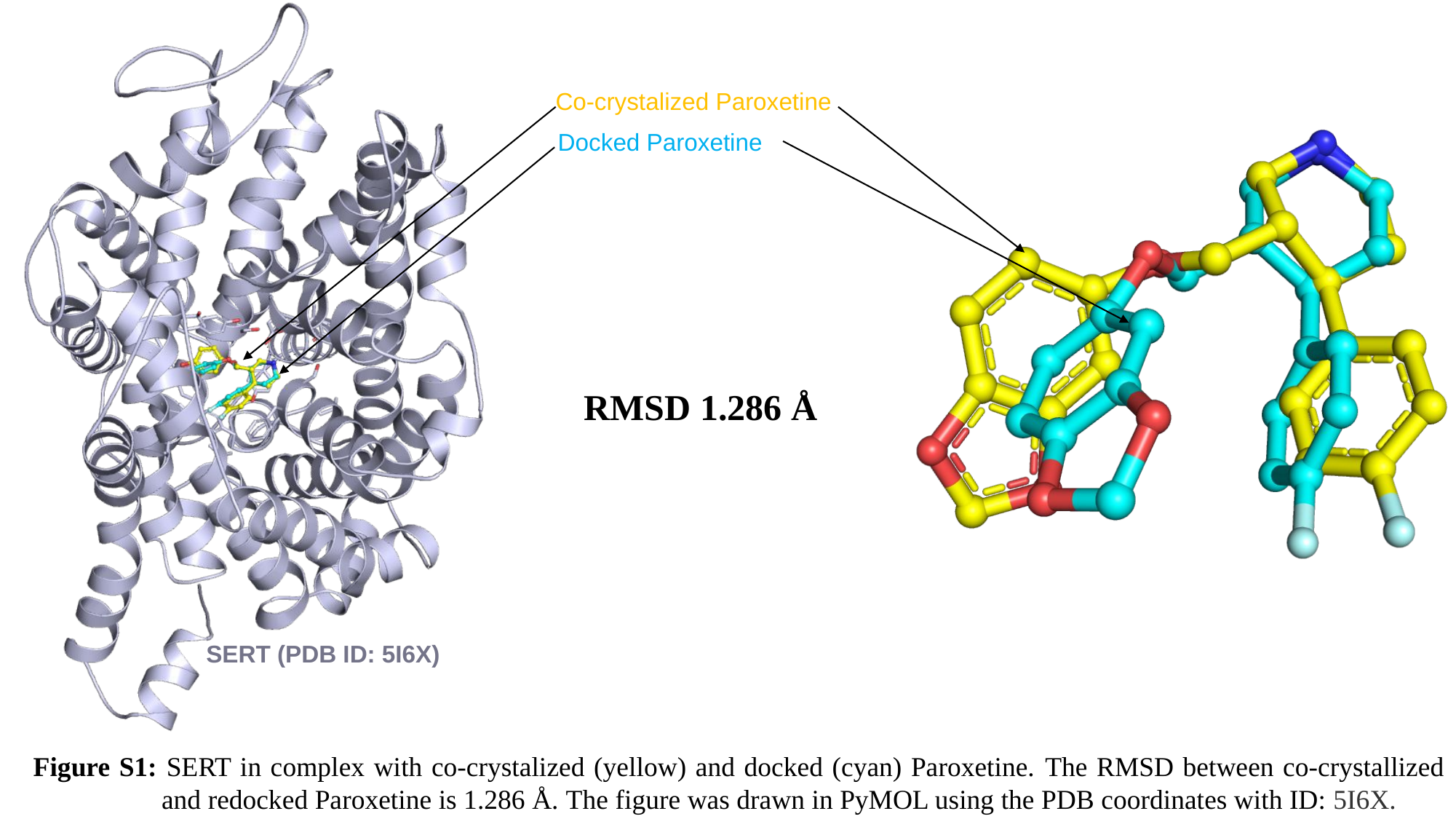

Co-crystalized Paroxetine
Docked Paroxetine
RMSD 1.286 Å
SERT (PDB ID: 5I6X)
Figure S1: SERT in complex with co-crystalized (yellow) and docked (cyan) Paroxetine. The RMSD between co-crystallized and redocked Paroxetine is 1.286 Å. The figure was drawn in PyMOL using the PDB coordinates with ID: 5I6X.

## Slide 2
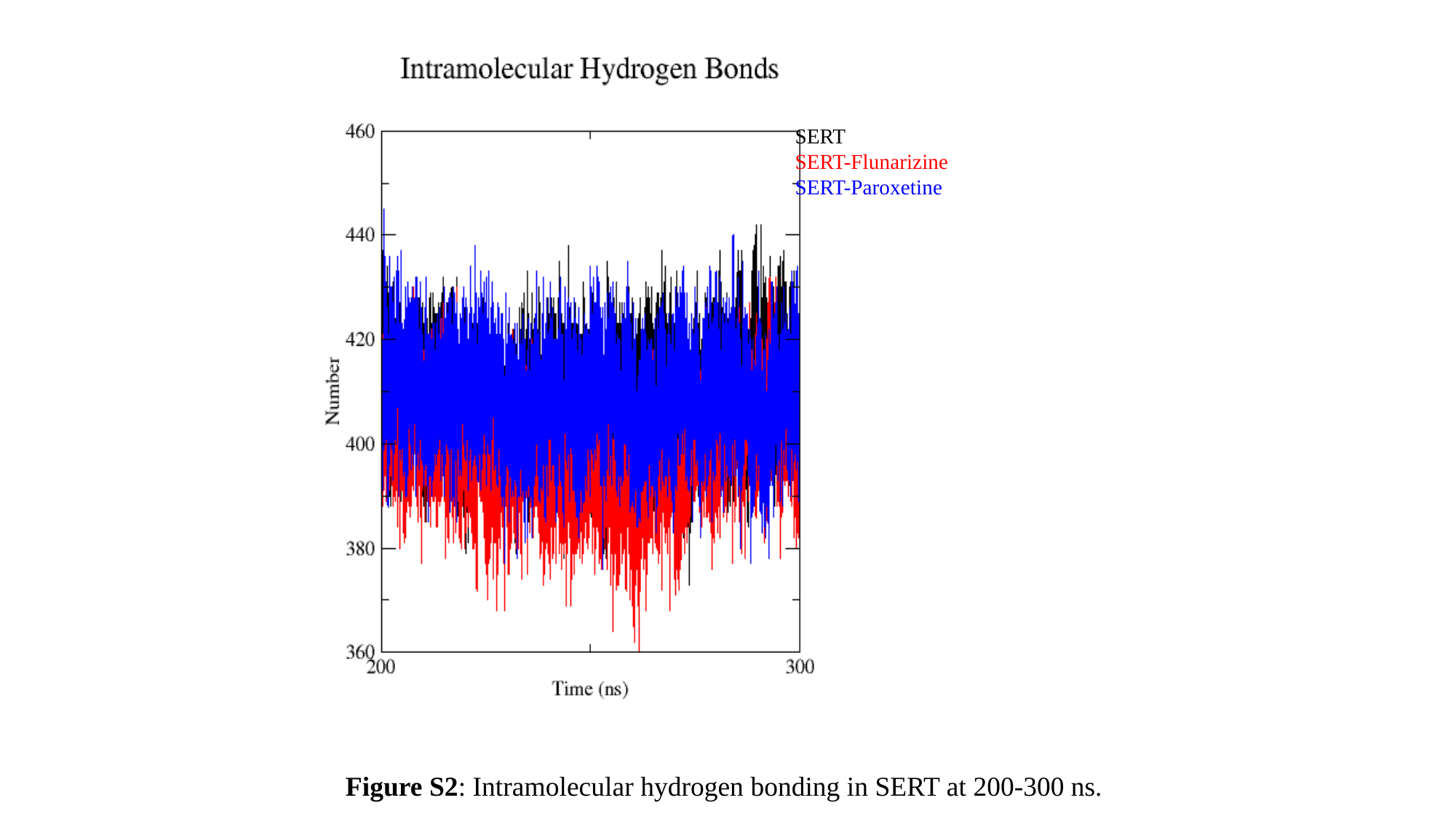

SERT
SERT-Flunarizine
SERT-Paroxetine
Figure S2: Intramolecular hydrogen bonding in SERT at 200-300 ns.

## Slide 3
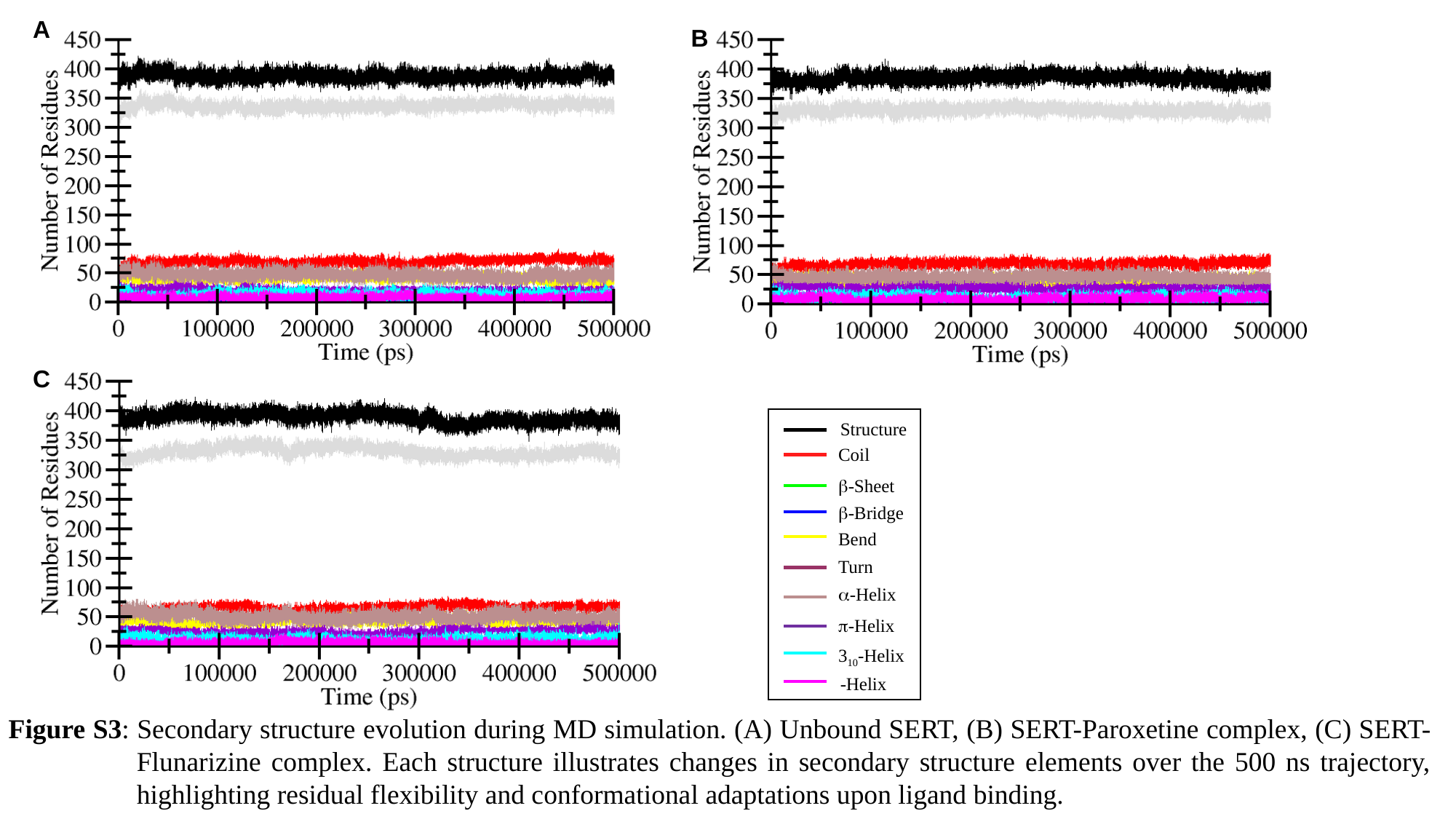

A
B
C
Structure
Coil
β-Sheet
β-Bridge
Bend
Turn
α-Helix
-Helix
310-Helix
Figure S3: Secondary structure evolution during MD simulation. (A) Unbound SERT, (B) SERT-Paroxetine complex, (C) SERT-Flunarizine complex. Each structure illustrates changes in secondary structure elements over the 500 ns trajectory, highlighting residual flexibility and conformational adaptations upon ligand binding.
